# Simplified S1 vertebral bone quality score independently predicts proximal junctional kyphosis after surgery for degenerative lumbar scoliosis

**DOI:** 10.1186/s13018-024-04722-y

**Published:** 2024-04-13

**Authors:** Wei Deng, Yue Zhou, Qingsong Zhou, Yong Yin, Yueming Song, Ganjun Feng

**Affiliations:** 1 Department of Orthopedics Surgery and Orthopedic Research Institute, West China Hospital, Sichuan University, Chengdu, 610041 China; 2 Department of Orthopedics, Pidu District People’s Hospital/The Third Affiliated Hospital of Chengdu Medical College, Chengdu, 611730 China; 3 Department of Emergency Medicine, West China Hospital, Sichuan University, Chengdu, China

**Keywords:** Proximal junctional kyphosis, Scoliosis, Vertebral bone quality, Bone mineral density

## Abstract

**Objective:**

Our study aimed to assess the effectiveness of the simplified S1 vertebral bone quality (VBQ) score in predicting the incidence of proximal junctional kyphosis (PJK) after surgery for degenerative lumbar scoliosis (DLS).

**Methods:**

We reviewed 122 patients with DLS who underwent posterior lumbar decompression and long-segment fusion surgery in our hospital from January 2016 to December 2020. The patients were classified into PJK group and non-PJK group. S1 VBQ scores are determined by signal intensity measurements taken from the mid-sagittal plane of T1-weighted non-contrast MRI. Logistic regression analysis was used to identify factors associated with PJK. Receiver-operating characteristic curve (ROC) analysis was used to evaluate the value of S1 VBQ score in predicting pedicle PJK after DLS.

**Results:**

122 DLS patients (90 females and 32 males) met the inclusion criteria. In addition, 27 patients (22.13%) had PJK at the time of last follow-up. VBQ was higher in PJK patients than non-PJK patients (3.58 ± 0.67 vs. 3.08 ± 0.54, *p* < 0.001). Preoperatively, patients in the PJK group had a greater TLK than those in the non-PJK group (20.00 ± 6.22 vs. 16.86 ± 5.38, *p* = 0.011). After surgery, patients in the PJK group had greater TLK (*p* < 0.001) and PJA (*p* < 0.001) compared with the non-PJK group. At final FU, patients in the PJK group had greater TK (*p* = 0.002), TLK (*p* < 0.001), SVA (*p* < 0.001), and PJA (*p* < 0.001) than patients in the non-PJK group (Table 4). In multivariate logistic regression analysis, higher VBQ score (OR 4.565, 95% CI 1.43–14.568, *p* = 0.010), advanced age (OR 1.119, 95% CI 1.021–1.227, *p* = 0.016), and larger TLK (OR 1.191, 95% CI 1.041–1.362, *p* = 0.011) were significant predictors of postoperative PJK in patients with DLS (Table 6). A statistically significant positive correlation existed between VBQ score and PJA change (r = 0.370, *p* < 0.001). We created ROC curves for VBQ scores as predictors of PJK with a diagnostic accuracy of 72.1% (95% CI 60.15–82.9%.The ideal limit for the VBQ score was 3.205 (sensitivity: 77.8%, specificity: 81.4%).

**Conclusion:**

To the best of our knowledge, this is the first study to evaluate the effectiveness of the S1 VBQ score in predicting postoperative PJK in DLS. Our study included major risk factors and found that S1 VBQ score was a significant predictor of PJK in patients undergoing DLS surgery. The higher the S1 VBQ score, the higher the probability of PJK.

**Supplementary Information:**

The online version contains supplementary material available at 10.1186/s13018-024-04722-y.

## Introduction

Degenerative lumbar scoliosis (DLS) is a common spinal disorder with a high incidence, especially in middle-aged and older adults [1, 2]. Although surgery is one of the effective methods to treat DLS, the incidence of postoperative proximal junctional kyphosis (PJK) remains high, posing significant challenges to patients 'rehabilitation and quality of life. Although the etiology of PJK is unknown, some studies suggest that osteopenia and osteoporosis may be associated with developing PJK after spinal corrective surgery [3, 4]. Osteopenia and osteoporosis can lead to instability of vertebral fixation and bone fusion, thereby increasing the risk of PJK [5, 6]. At present, dual-energy X-ray absorptiometry (DEXA) is widely regarded as the gold standard for measuring bone mineral density [7]. However, DEXA is susceptible to interference from spinal degeneration, which makes the bone quality in the repair area imprecise.

In recent years, vertebral bone quality (VBQ) score using non-contrast T1-weighted lumbar magnetic resonance imaging (MRI) has emerged as a new technique for assessing bone quality [8, 9]. Studies have shown that VBQ score is a more accurate predictor of osteoporosis than DEXA [10, 11]. However, it is challenging for some patients to obtain signal intensity (SI) for the L1-4 vertebral in the mid-sagittal plane due to scoliosis. Huang et al. [9] modified and simplified the traditional L1-4 VBQ measurement method with the VBQ of S1 vertebral body and demonstrated the potential feasibility of this method in clinical practice. In this study, we initially assessed the effectiveness of the simplified S1 VBQ score in predicting the incidence of PJK in patients with DLS.

## Patients and methods

The present work is a single-center retrospective study. Retrospective analysis of 122 patients with DLS who underwent posterior lumbar decompression and long-segment fusion surgery in our hospital from January 2016 to December 2020. Inclusion criteria: (1) age ≥ 50 years; (2) level of fusion ≥ 4, and upper instrumented vertebrae (UIV) located in the thoracolumbar segment; (3) follow-up ≥ 24 months; (4) preoperative T1-weighted non-contrast MRI scan; (5) Clinical data and imaging data were complete. Exclusion criteria included: (1) idiopathic scoliosis, neuromuscular scoliosis, and nonstructural scoliosis; (2) spinal tumor, spinal tuberculosis, and spinal infectious disease; (3) history of spinal trauma, spinal surgery, and hip surgery; (4) patients with chronic liver disease, renal failure, and metabolic bone disease other than osteopenia or osteoporosis.

### VBQ

**Fig. 1:**
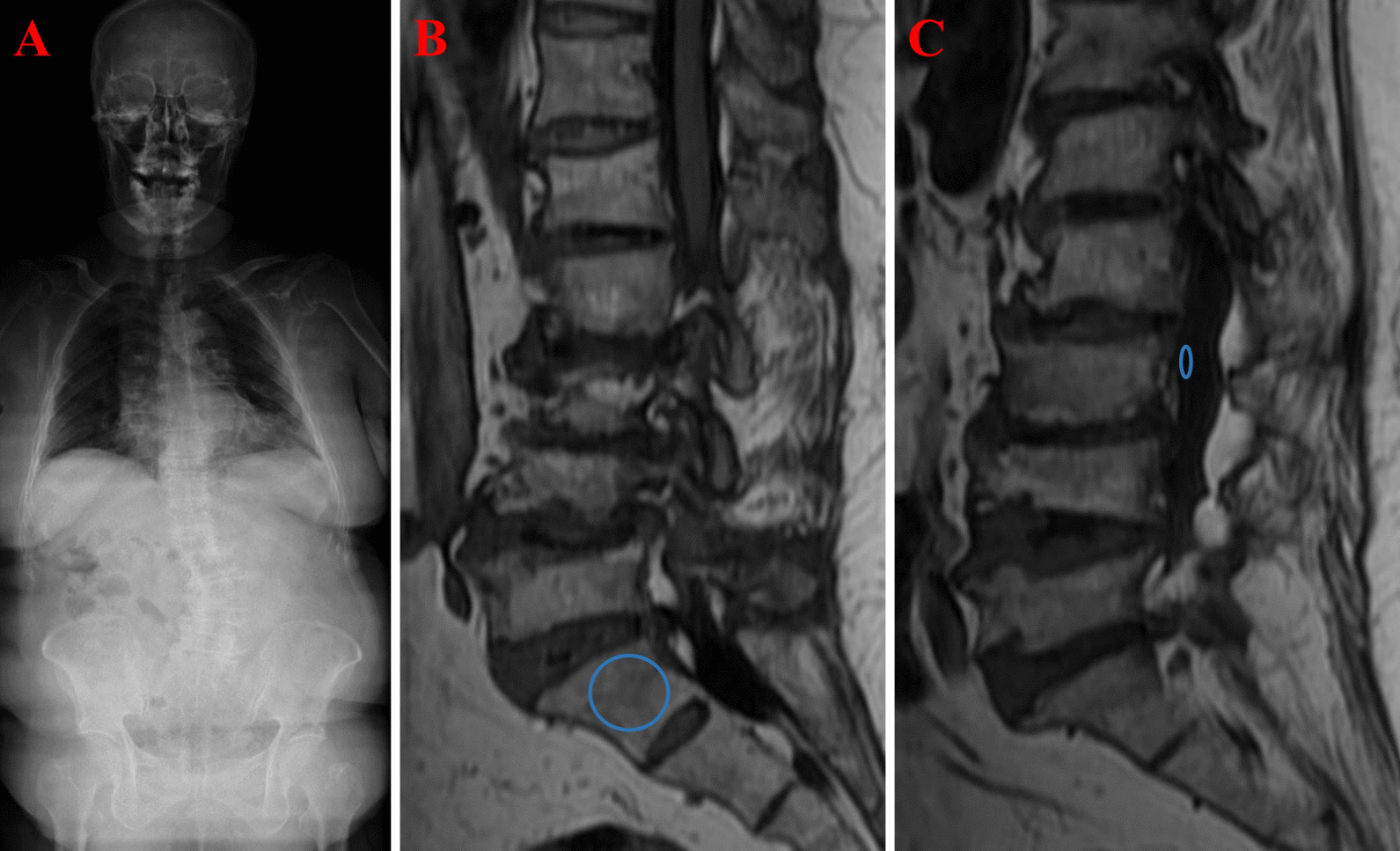
**A** A typical standing full-length posteroanterior plain radiography of DLS clearly showed the structural lumbar curve (**B**, **C**). Sagittal non-contrast-enhanced T1-weighted MRI of the lumbar spine detailing the SI of the ROI (circles) used to calculate the S1 VBQ score

As previously described in the literature [8], S1 VBQ scores are determined by SI measurements taken from the mid-sagittal plane of T1-weighted non-contrast MRI. Select an elliptical region of interest (ROI) as significant as possible in the mid-sagittal plane of each MRI image, covering the skeletal region of the S1 vertebral body, avoiding lesions including hemangiomas or Schmorl’s nodes, and automatically calculate the average SI within the ROI. This average SI value is then divided by the average SI value of the selected CSF region in the sagittal plane in L3 of the same patient. The S1 VBQ score is then calculated similarly to the L1-4 VBQ score (Fig. 1).

$${\text{S}}1{\text{ VBQ}} = {{{\text{SI}}_{{{\text{S}}1}} } \mathord{\left/ {\vphantom {{{\text{SI}}_{{{\text{S}}1}} } {{\text{SI}}_{{{\text{CSF}}}} }}} \right. \kern-0pt} {{\text{SI}}_{{{\text{CSF}}}} }}$$

### Radiographic measurements and clinical outcomes

All patients had anteroposterior and lateral spine radiographs taken preoperatively, postoperatively, and at the last follow-up. Two experienced spine surgeons performed all measurements, and the results averaged over two different times. Measurement parameters include: (1) thoracic kyphosis angle (TK), defined as the angle between the superior endplate of T5 and the inferior endplate of T12; (2) thoracolumbar kyphosis angle (TLK), defined as the angle between the superior endplate of T10 and the inferior endplate of L2; (3) lumbar lordosis angle (LL), defined as the angle between the superior endplate of L1 and the superior endplate of S1; (4) Pelvic incidence angle (PI), defined as the angle between the line passing through the midpoint of S1 upper endplate and the midpoint of the line passing through the midpoint of S1 upper endplate and the center of both femoral heads, and the vertical line of S1 upper endplate; (5) Pelvic inclination angle (PT), defined as the angle between the line passing through the midpoint of S1 upper endplate and the midpoint of the line passing through the midpoint of S1 upper endplate and the center of both femoral heads, and the vertical line; (6) Sacral inclination angle (SS), defined as the angle between S1 upper endplate and the horizontal line; (7) sagittal balance (SVA), defined as the vertical distance between the C7 plumb line and the posterior superior angle of the sacrum, positive values indicate that the C7 plumb line is anterior to the posterior superior angle of the sacrum, negative values indicate posterior; (8) Proximal junctional angle (PJA), which indicates the angle between the upper endplate of UIV + 2 and the lower endplate of UIV; (9) PI-LL, defined as the difference between PI and LL, i.e. PI-(-LL). PJK was defined by a change in the proximal junctional angle greater than or equal to 10 between the immediate postoperative and the final follow-up radiograph. Routine follow-up included whole-spine anteroposterior and lateral X-rays at 1 month, 3 months, 6 months, and annually thereafter. At postoperative follow-up, clinical outcomes were assessed using Japanese Orthopaedic Association (JOA) scores, Oswestry Disability Index (ODI), and Visual Analogue Scale (VAS). These scoring tools are widely used to assess the functional status of spinal disorders, and they are practical and rigorous measures of functional assessment.

### Consistency evaluation

First, the investigators randomly selected 60 patients from the sample for repeated measurements to assess intra-observer reliability. Subsequently, two independent investigators randomly selected another 60 patients for VBQ measurements to assess inter-observer reliability. Intra-observer and inter-observer reliability were evaluated using the intra-class correlation coefficient (ICC). According to the Fleiss guidelines, the interpretation of ICC values is as follows: below 0.40 indicates poor confidence, between 0.40 and 0.75 indicates fair confidence, and above 0.75 indicates good confidence.

### Statistical analysis

Continuous variables are expressed as mean ± standard deviation, and categorical variables are expressed as frequencies and/or percentages. The independent or paired sample t-test is used for the measurement data with normal distribution. The independent or paired sample Mann–Whitney U test is used for the measurement data that do not conform to normal distribution. Statistical data were compared by Chi-square test or Fisher test. A multivariate logistic regression model was used to include all clinically relevant risk factors. The receiver operating characteristic (ROC) curve and area under the curve (AUC) were used to determine the separation criteria between PJK group and non-PJK group and to establish a VBQ score threshold (cut-off value) with high sensitivity and specificity. The correlation between PJA and VBQ scores was assessed using one-way linear regression. All data were analyzed using SPSS 23.0 (SPSS, IBM Analytics, New York, USA) software. *p* < 0.05 was considered statistically significant.

## Results

Intra-observer and inter-observer reliability between two spine surgeons were almost perfect. The ICC value of variables were all greater than 0.75, which indicated an excellent reliability (Additional file 1: Table S1).

A total of 122 DLS patients met the inclusion criteria (90 females and 32 males). In addition, 27 patients (22.13%) had PJK at the time of last follow-up. In all patients followed up, PJK was diagnosed by routine imaging at a mean of 5.9 months (range 3–27 months) postoperatively. VBQ was higher in PJK patients than non-PJK patients (3.58 ± 0.67 vs. 3.08 ± 0.54, *p* < 0.001). There were no significant differences in age (*p* = 0.063), gender (*p* = 0.052), BMI (*p* = 0.064), LIV to sacrum (*p* = 0.423), T score (*p* = 0.236), Levels of fusion (*p* = 0.248), and follow-up time (*p* = 0.637) between the two groups (Table 1). Table 1 summarizes demographic and procedural data.

**Table 1:** Comparisons of demographic and surgical data between the DLS patients with and without PJK

Variables	PJK (n = 27)	No PJK (n = 95)	*p* value
Age, mean (SD)	64.33 ± 9.02	60.97 ± 7.99	0.063
Gender, N (%)			0.052
Male	11 (40.7)	21 (22.1)	
Female	16 (59.3)	74 (77.9)	
BMI, mean (SD)	26.72 ± 4.18	25.19 ± 3.61	0.064
Smoker, N (%)			0.264
Yes	5 (18.5)	10 (10.5)	
No	22 (81.5)	85 (89.5)	
Alcohol abuse, N (%)			0.521
Yes	6 (22.2)	16 (16.8)	
No	21 (77.8)	79 (83.2)	
Hypertension, N (%)			0.789
Yes	11 (40.7)	36 (37.9)	
No	16 (59.3)	59 (62.1)	
Diabetes, N (%)			0.537
Yes	4 (14.8)	10 (10.5)	
No	23 (85.2)	85 (89.5)	
LIV to sacrum			0.423
Yes	16 (59.3)	48 (50.5)	
No	11 (40.7)	47 (49.5)	
T score, mean (SD)	− 1.96 ± 1.29	− 1.59 ± 1.46	0.236
VBQ, mean (SD)	3.58 ± 0.67	3.08 ± 0.54	< 0.001
Levels of fusion, mean (SD)	6.48 ± 1.45	6.12 ± 1.44	0.248
Mean follow-up, months	48.96 ± 19.90	50.82 ± 17.46	0.637

Preoperatively, patients in the PJK group had a greater TLK than those in the non-PJK group (20.00 ± 6.22 vs. 16.86 ± 5.38, *p* = 0.011). A comparison of preoperative imaging parameters between the two groups is shown in Table 2. After surgery, patients in the PJK group had greater TLK (*p* < 0.001) and PJA (*p* < 0.001) compared with the non-PJK group. A comparison of postoperative imaging parameters between the two groups is shown in Table 3. At final FU, patients in the PJK group had greater TK (*p* = 0.002), TLK (*p* < 0.001), SVA (*p* < 0.001), and PJA (*p* < 0.001) than patients in the non-PJK group (Table 4).

**Table 2:** Comparisons of radiographic parameters between the DLS patients with and without PJK before surgery

Variables	PJK (n = 27)	No PJK (n = 95)	*p* value
Preoperative TK	20.04 ± 10.72	18.03 ± 12.54	0.452
Preoperative TLK	20.00 ± 6.22	16.86 ± 5.38	0.011
Preoperative LL	26.01 ± 11.68	22.60 ± 15.19	0.217
Preoperative SS	19.46 ± 11.50	23.21 ± 10.01	0.099
Preoperative PI	50.91 ± 11.04	48.21 ± 10.01	0.229
Preoperative PT	27.89 ± 11.35	26.15 ± 10.59	0.458
Preoperative SVA	47.27 ± 32.46	44.68 ± 35.90	0.737
Preoperative PI-LL	24.89 ± 8.67	26.29 ± 12.89	0.514
Preoperative PJA	6.23 ± 4.37	6.33 ± 3.55	0.915

**Table 3:** Comparisons of radiographic parameters between the DLS patients with and without PJK postoperatively

Variables	PJK (n = 27)	No PJK (n = 95)	*p* value
Postoperative TK	25.04 ± 10.78	22.59 ± 12.78	0.366
Postoperative TLK	16.60 ± 5.79	10.94 ± 5.74	< 0.001
Postoperative LL	34.10 ± 13.15	31.38 ± 15.73	0.412
Postoperative SS	25.60 ± 9.65	25.98 ± 10.07	0.859
Postoperative PI	51.28 ± 11.05	48.47 ± 9.94	0.208
Postoperative PT	23.88 ± 11.02	22.08 ± 10.39	0.435
Postoperative SVA	40.51 ± 28.57	34.54 ± 28.38	0.428
Postoperative PI-LL	18.19 ± 9.33	20.27 ± 11.64	0.408
Postoperative PJA	11.21 ± 4.09	7.42 ± 4.36	< 0.001

**Table 4:** Comparisons of radiographic parameters between the DLS patients with and without PJK at final follow-up

Variables	PJK (n = 27)	No PJK (n = 95)	*p* value
TK at last follow-up	32.41 ± 9.46	25.17 ± 12.74	0.002
TLK at last follow-up	21.47 ± 6.16	12.51 ± 2.41	< 0.001
LL at last follow-up	31.92 ± 13.55	28.26 ± 16.17	0.285
SS at last follow-up	21.15 ± 9.88	23.97 ± 10.16	0.203
PI at last follow-up	51.76 ± 11.09	48.95 ± 9.94	0.209
PT at last follow-up	26.60 ± 11.11	24.54 ± 10.56	0.380
SVA at last follow-up	65.33 ± 17.76	45.53 ± 33.53	< 0.001
PI-LL at last follow-up	20.72 ± 9.21	23.44 ± 11.76	0.281
PJA at last follow-up	21.13 ± 8.35	9.79 ± 4.92	< 0.001

At baseline, the two groups had no difference in VAS, JOA, ODI scores. At final follow-up, VAS-back, VAS-leg, and JOA scores were worse in the PJK group than in the non-PJK group (Table 5).

**Table 5:** Comparisons of clinical parameters between the DLS patients with and without PJK

Variables	PJK (n = 27)	No PJK (n = 95)	*p* value
VAS (back)			
Preoperative	4.89 ± 1.28	5.12 ± 1.24	0.405
At last follow-up	3.03 ± 0.97	2.34 ± 0.86	0.001
VAS (leg)			
Preoperative	4.78 ± 1.32	5.04 ± 1.46	0.405
At last follow-up	2.92 ± 0.74	1.99 ± 1.01	< 0.001
JOA			
Preoperative	13.52 ± 3.39	13.58 ± 3.43	0.941
At last follow-up	18.08 ± 3.46	22.44 ± 5.23	< 0.001
ODI			
Preoperative	48.65 ± 11.61	49.42 ± 11.84	0.764
At last follow-up	29.10 ± 5.58	26.85 ± 13.50	0.202

**Fig. 2:**
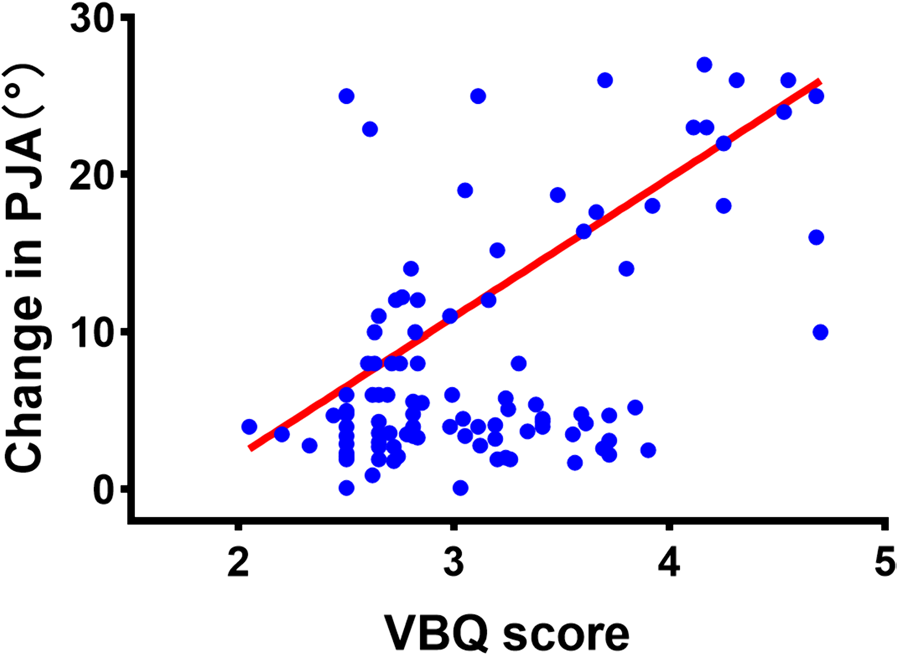
Graph showing a positive, significant correlation between PJA (°) change and S1 VBQ score

In multivariate logistic regression analysis, higher VBQ score (OR 4.565, 95% CI 1.43–14.568, *p* = 0.010), advanced age (OR 1.119, 95% CI 1.021–1.227, *p* = 0.016), and larger TLK (OR 1.191, 95% CI 1.041–1.362, *p* = 0.011) were significant predictors of postoperative PJK in patients with DLS (Table 6). A statistically significant positive correlation existed between VBQ score and PJA change (r = 0.370, *p* < 0.001; Fig. 2).

**Table 6:** Multivariate logistic regression analysis of risk factors for PJK

Variables	Odds ratio	95% CI	*p* value
VBQ	4.565	1.43, 14.568	0.010
T score	1.001	0.588, 1.704	0.998
age	1.119	1.021, 1.227	0.016
Sex	4.095	0.844, 19.868	0.080
BMI	1.214	0.986, 1.495	0.068
Fused levels	0.84	0.485, 1.457	0.535
LIV to sacrum	0.331	0.075, 1.451	0.142
Preoperative TK	0.993	0.926, 1.064	0.835
Preoperative TLK	1.191	1.041, 1.362	0.011
Preoperative LL	1.967	1.661, 2.745	0.794
Preoperative SS	0.41	0.088, 1.604	0.999
Preoperative PI	0.005	0.001, 0.045	0.993
Preoperative PT	0.957	0.833, 1.099	0.529
Preoperative SVA	1.001	0.976, 1.024	0.978
Preoperative PI-LL	1.202	1.018, 1.477	0.794
Preoperative PJA	0.973	0.803, 1.179	0.779

**Fig. 3:**
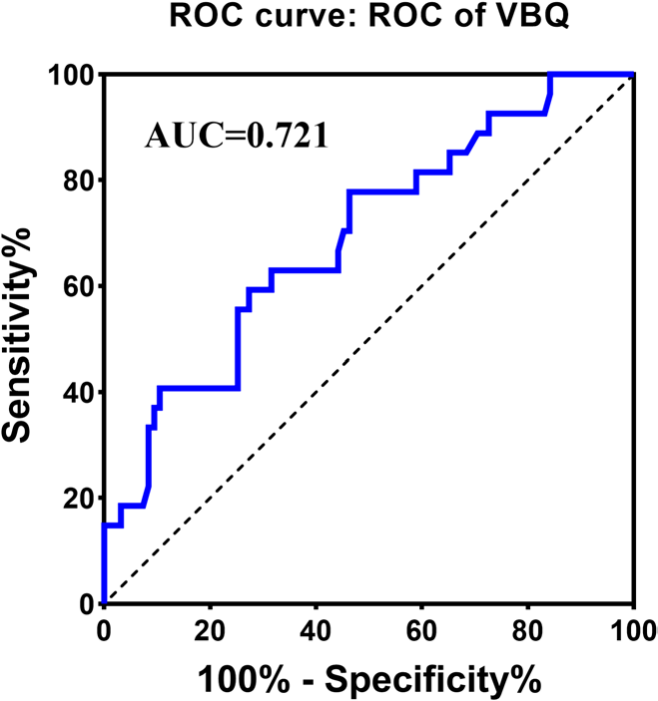
ROC curve for the multivariate model using S1 VBQ score as a predictor of proximal junctional kyphosis after surgery for DLS

We created ROC curves for VBQ scores as predictors of PJK with a diagnostic accuracy of 72.1% (95% CI 60.15–82.9%; Fig. 3). The ideal limit for the VBQ score was 3.205 (sensitivity: 77.8%, specificity: 81.4%).

## Discussion

PJK is the most common complication after DLS surgery, which can lead to progressive sagittal decompensation, nerve damage, revision surgery, and worse clinical outcomes [12, 13]. The incidence of PJK varies from one literature report to another but can typically reach between 10 and 30% within 12 months of surgery [14–16]. In our study, PJK was observed in 27 patients with a prevalence of 22.13%, which is similar to what Wang et al. [17, 18] reported in patients with DLS.

The development and progression of PJK are associated with multiple factors, as no study has shown that a single etiology is closely and consistently associated with their development. Relevant studies have identified risk factors associated with patients, procedures, and imaging. In this study, preoperative TK did not differ significantly between PJK and non-PJK groups. It was mentioned that there is no consensus on standard values for TK and that thoracic compensatory capacity may manifest as kyphosis and degenerative spine. Meanwhile, the incidence of TK for PJK before surgery is controversial in the literature. Oe et al. [19] pointed out that both kyphosis and kyphosis excess of TK preoperatively contributed to the high incidence of PJK. Our results found that larger TLK (OR 1.191, 95% CI 1.041–1.362, *p* = 0.011) was an independent risk factor for postoperative PJK in patients with DLS. According to previous literature [20, 21], PJK may be a compensatory mechanism resulting from changes in spinal balance, and patients with higher TLK are at greater risk for PJK. Greater TLK may lead to sagittal imbalance, and the occurrence of PJK after corrective surgery is a compensatory mechanism of sagittal imbalance, consistent with our findings. Age, BMI, and osteoporosis were considered risk factors associated with patients. Kim et al. [22] also found older patients with PJK requiring revision. However, in our study, age and BMI were similar. A decrease in bone mineral density (BMD) is another crucial risk factor for PJK and can lead to loosening of pedicle screws and compression fractures in UIV.

DEXA is currently considered the gold standard for osteoporosis screening. However, due to osteophyte proliferation, the accuracy of traditional DEXA measurements for certain degenerative diseases has also been questioned. Factors such as degenerative changes in the lumbar spine or superimposed calcified tissue may cause bone mineral density in patients with osteoporosis to be incorrectly overestimated, influencing surgeons to make the best decisions. Computed tomography-based Hounsfeld unit (HU) measurements have been reported as an alternative method for accurately assessing bone mass. Pickhardt et al. [23] confirmed the diagnostic value of L1 HUs, while Zou et al. [24] further evaluated the use of S1 HUs, providing a simple method for directly assessing S1 BMD.

Recently, MRI-based bone mass measurements have increasingly been applied to lumbar spine BMD assessment to reduce patient radiation exposure [25–27]. MRI bone analysis shows that when vertebral BMD decreases, trabecular bone increases due to fat infiltration and higher signal on T1-weighted imaging. This method has been shown to accurately assess vertebral bone mass, an essential predictor of osteopenia and osteoporosis. Kuo et al. [28] reported that the higher VBQ was independently associated with PJK in patients undergoing DLS correction. VBQ score measured by preoperative MRI may be a valuable adjunct to DLS surgical planning. However, accurate measurement of VBQ score using conventional measurement methods is variable due to the lumbar spine profile of DLS. Therefore, Huang et al. [26] proposed a new simplified S1 VBQ score concerning the measurement method of S1 HU. The results showed that the diagnostic accuracy of S1 VBQ method was comparable to the results of L1-4 VBQ or S1 HU, and the measurement method was more straightforward to ensure reliability within/among raters. The S1 VBQ score is a promising alternative to assessing BMD in patients with DLS. As in previous studies, our results show that S1 VBQ score is an independent risk factor for PJK after DLS, and S1 VBQ score is a good predictor of PJK (AUC = 0.721). The ideal limit of VBQ score was 3.205 (sensitivity: 77.8%, specificity: 81.4%), indicating that VBQ score is still a very sensitive parameter for assessing PJK risk after excluding other factors.

At present, the choice of distal fixation fusion segment, whether fusion to S1 or not, is still the focus of debate. LIV located at L5 can preserve more motor function of the active segment and improve the quality of life of patients. However, fusion to L5 alone accelerates degeneration of the L5-S1 disc, which may lead to further scoliosis and sagittal imbalance. Fusion to S1 results in the loss of L5-S1 motion segments and changes in spinal-pelvic biomechanics, which may exacerbate sacroiliac joint pain and accelerate sacroiliac joint degeneration. Furthermore, fusion to the sacrum increases surgical exposure, prolongs surgical time, and may increase the incidence of complications. Liu et al. [29] conducted a meta-analysis of 14 studies and showed that fusion to the sacrum was a risk factor for PJK. Yagi et al. [13] also found that the incidence of PJK was twice as high in patients with LIV fixation to the sacrum as in patients with LIV fixation at the superior sacrum. Surgical risk factors should also consider factors such as the number of fusion levels and interbody fusion. Bridwell et al. [30] found that too short fusion segment is also a risk factor for PJK. In this study, by comparing the surgical data of PJK group and non-PJK group, it was found that the fixed position, fixed level and interbody fusion of UIV and LIV were not risk factors for PJK. Due to the small sample size of this study, further accumulation of cases needs to be further analyzed.

### Limitations

The study also has some limitations. First, this is a single-center retrospective study with a relatively limited sample size, and further, more extensive multicenter studies are needed to help validate and reinforce these findings. Our analysis did not include preoperative laboratory values such as calcium, phosphorus, glomerular filtration rate, and bone metabolism, leading to a more comprehensive assessment of osteoporosis risk. This work will help improve the potential complementary role of S1 VBQ in predicting osteoporosis.

## Conclusion

Higher VBQ score, advanced age, and larger TLK were significant predictors of postoperative PJK in patients with DLS. Our study is the first to assess the prognostic utility of S1-based VBQ score in predicting the occurrence of PJK after DLS. VBQ score could significantly predict the occurrence of PJK with an accuracy of 72.1%. S1 VBQ score assessment may be an effective alternative to the classical VBQ score and is expected to advance bone quality assessment and PJK prediction in patients with DLS.

### Supplementary Information

**Additional file: Table S1.** The ICC values of quantitative parameters between two spine surgeons.

## Data Availability

Data will be available upon request to the corresponding author.

## Abbreviations

VBQ: Vertebral bone quality
MRI: Magnetic resonance imaging
PJK: Proximal junctional kyphosis
DLS: Degenerative lumbar scoliosis
TK: Thoracic kyphosis angle
TLK: Thoracolumbar kyphosis angle
LL: Lumbar lordosis angle
PI: Pelvic incidence angle
PT: Pelvic inclination angle
UIV: Upper instrumented vertebral body
BMD: Bone mineral density
DEXA: Dual-energy X-ray absorptiometry
ICC: Intra-class correlation coefficient
BMI: Body mass index
ROI: Regions of interest
CSF: Cerebrospinal fluid
SI: Signal intensity
ROC: Receiver operating characteristic
AUC: Area under the curve
SS: Sacral inclination angle
SVA: Sagittal balance
PJA: Proximal junctional angle
JOA: Japanese Orthopaedic Association
ODI: Oswestry disability index
VAS: Visual analogue scale
