# Supplementary material for: Simplified S1 vertebral bone quality score independently predicts proximal junctional kyphosis after surgery for degenerative lumbar scoliosis
Source: J Orthop Surg Res. 2024 Apr 13;19:238. doi: 10.1186/s13018-024-04722-y (PMC11015578; doi:10.1186/s13018-024-04722-y)
Supplement: Supplementary file 1 — Additional file: Table S1. The ICC values of quantitative parameters between two spine surgeons. [file 13018_2024_4722_MOESM1_ESM.docx]

| **Supplemental Table 1. The ICC values of quantitative parameters between two** spine surgeons. | | |
| --- | --- | --- |
| **Characteristics** | **Intraobserver agreement** | **Interobserver agreement** |
|  | **ICC value** | **ICC value** |
| TK | 0.905(0.749, 0.974) | 0.882(0.852, 0.943) |
| TLK | 0.913(0.894, 0.956) | 0.893(0.861, 0.942) |
| LL | 0.933(0.904, 0.977) | 0.901(0.887, 0.958) |
| SS | 0.946(0.915, 0.986) | 0.931(0.902, 0.997) |
| PI | 0.895(0.867, 0.951) | 0.835(0.802, 0.884) |
| PT | 0.923(0.903, 0.966) | 0.897(0.828, 0.944) |
| SVA | 0.948(0.917, 1.000) | 0.933(0.902, 0.977) |
| PJA | 0.883(0.833, 0.906) | 0.826(0.800, 0.887) |
